# A novel efficient drug repurposing framework through drug-disease association data integration using convolutional neural networks

**DOI:** 10.1186/s12859-023-05572-x

**Published:** 2023-11-22

**Authors:** Ramin Amiri, Jafar Razmara, Sepideh Parvizpour, Habib Izadkhah

**Affiliations:** 1 Department of Computer Science, Faculty of Mathematics, Statistics and Computer Science, University of Tabriz, Tabriz, Iran; 2 Research Center for Pharmaceutical Nanotechnology, Biomedicine Institute, Tabriz University of Medical Sciences, Tabriz, Iran; 3 Department of Medical Biotechnology, Faculty of Advanced Medical Sciences, Tabriz University of Medical Sciences, Tabriz, Iran

**Keywords:** Drug repurposing, Data integration, Machine learning, Deep learning

## Abstract

Drug repurposing is an exciting field of research toward recognizing a new FDA-approved drug target for the treatment of a specific disease. It has received extensive attention regarding the tedious, time-consuming, and highly expensive procedure with a high risk of failure of new drug discovery. Data-driven approaches are an important class of methods that have been introduced for identifying a candidate drug against a target disease. In the present study, a model is proposed illustrating the integration of drug-disease association data for drug repurposing using a deep neural network. The model, so-called IDDI-DNN, primarily constructs similarity matrices for drug-related properties (three matrices), disease-related properties (two matrices), and drug-disease associations (one matrix). Then, these matrices are integrated into a unique matrix through a two-step procedure benefiting from the similarity network fusion method. The model uses a constructed matrix for the prediction of novel and unknown drug-disease associations through a convolutional neural network. The proposed model was evaluated comparatively using two different datasets including the gold standard dataset and DNdataset. Comparing the results of evaluations indicates that IDDI-DNN outperforms other state-of-the-art methods concerning prediction accuracy.

## Introduction

Drug repurposing means a new use of a drug other than its original and approved use [1]. In recent years, drug repurposing has attracted the attention of most pharmaceutical companies regarding cost reduction and low failure rate compared to traditional drug production methods. Drug repurposing can be useful in identifying new, low-cost, and short-time treatments for diseases for which preclinical safety studies have been completed. The development of traditional treatment methods to produce a new treatment solution takes nearly 17 years, and its rate of success is less than 10% [2]. Therefore, there is a huge and significant need to produce new medications for diseases for which drugs result in side effects and unpleasant effects for patients i.e. emerging ones such as COVID-19, which brings the whole world into a fundamental challenge, and rare diseases. Recent research reports show that there exist about seven thousand rare diseases that have no effective treatment, which imposes their effect on more than 400 million people worldwide [3–5].

In recent years, researchers have conducted studies on drug repurposing. These studies have been mostly on the analysis and description of drug repurposing methods along with their successful examples. Successful examples of drug repurposing include Sildenafil (Viagra), which was previously used to treat erectile dysfunction, but now new uses have been discovered for it using repurposing. Bupropion, which is generally used to treat depression, is now also used for smoking cessation and thalidomide, which was introduced for the treatment of morning sickness, is now recommended for multiple myeloma [2, 6–12].

Through computational methods based on association analysis between a pair of drug-disease (DD), one can predict new applications of those range of known drugs used previously. In addition, related reliability has also been proven experimentally. Two categories of such methods for assessing computational experiments are worth drawing attention to; one category is based on the drug-disease relationship, which acts based on the common protein or gene complex between the drug and the disease. Another category, in addition to drugs, diseases, and target associations, also benefits from the in-between similarity. Drug repurposing studies generally concentrate on discovering similarities in drug mode of action [13], revealing new drug indications [14], investigating common features among drug combinations [15], and discovering drug-disease relationships [7]. The major challenge of this kind of study is the identification of the real target molecule of a certain drug among hundreds of thousands of additional genes that indirectly affect the results of the studies. Classic statistical models and approaches are not effective for discovering and distinguishing the target molecule of a certain drug among thousands of genes.

Furthermore, the major drawback of many traditional repurposing methods is the use of one source of data, because in this way only a specific part of the behavioral knowledge of a living organism is examined. Also, the same methods suffer missing and incorrect data affecting their performance. For example, numerous reasons are involved in imposing difficulty in defining profiles of gene expression signatures reliably. Moreover, when using these genes as drug targets significant changes in gene expression may not always occur, leading to inaccurate data. In addition, the lack of clear data for target drugs when using the chemical structure and molecular information makes it difficult to identify associations of drug targets. As a result, claims of inference and discovery regarding the mentioned methods may be unsustainable. Therefore, the integration of data from various sources during the development of repurposing computational models overcomes this challenge [16–18].

Most information-based approaches to detecting drug-disease association patterns obtain their data from patients, healthcare professionals, and pharmaceutical companies [19–24]. Also, in recent years, the efforts of researchers have gone towards predictive models such as machine learning (ML), which are used to discover drug-disease associations during the drug production process or before the commercial introduction of the drug in the market [20]. The two main machine learning approaches for this purpose are network-based [25–27] and similarity-based [28–34]. Network-based ML methods have been introduced recently to predict drug-disease associations. Because they are capable of extracting and integrating knowledge of multiple information sources such as chemical, biological, target, genomic, and pharmaceutical sources. In 2022, Zhao et al*.* [35] proposed a geometric deep learning (GDL) framework, namely DDAGDL, to predict drug-disease associations (DDAs) on heterogeneous information networks (HINs). DDAGDL can take to learn the feature representations of drugs and diseases by ingeniously projecting drugs and diseases including geometric prior knowledge of network structure in a non-Euclidean domain onto a latent feature space. The model suggests new high-quality drugs for Alzheimer’s disease and Breast neoplasms. The results of evaluations in terms of accuracy, recall, precision, and F1-score were 0.842, 0.849, 0.836, and 0.843, respectively. In 2023, Zhao et al*.* [36] proposed a graph learning-based method by integrating the biological knowledge of drugs and targets with their interactions. They used a gradient-boosting decision tree classifier to predict novel drug-target associations. They obtained a high performance in their evaluations in terms of AUC, AUPR, and F1-score equal to 0.965, 0.967, and 0.899, respectively.

Some network-based ML methods create a drug-target network and discover drug-disease associations using the strength of network connections or by recognizing drug pairs that share drug targets or drug pathways [25–27]. In 2013, Cami et al. [37] proposed a drug association network to predict drug-disease associations using the network's topological structure for all known associations. Based on the drug's intrinsic and taxonomic properties, the PPIN reports a sensitivity of 48%, a specificity of 90%, and an area under the receiver operating characteristic curve (AUROC) of 81%.

Machine learning methods based on similarity for predicting drug-disease associations mostly use binary classification. The binary similarity measurements vary based on adding and subtracting negative matches. In addition, some criteria consider both positive and negative weighted matches to obtain optimal performance. Hamming-based, correlation-based, and inner product-based methods are the main criteria of binary similarity [28, 30–33]. For improving prediction performance for DD association, in the present research, we propose a computational method that makes use of molecular characteristics as well as multiple similarities related to drugs and diseases. This method called IDDI-DNN (Integration of Drug-Disease associations for drug repurposing by Deep Neural Network) integrates multiple similarities between drugs and diseases and employs deep neural networks to capture similarities between them. The method first integrates multiple data related to drugs, diseases, and drug-disease associations into a unique similarity matrix during three steps, and then, uses the constructed matrix to train a convolutional neural network (CNN). The model is used to suggest a suitable drug for a target disease. Relying on the results of conducted experiments, IDDI-DNN outperforms several state-of-the-art methods through the use of benchmark datasets in terms of Receiver Operating Characteristic (ROC) and Precision-Recall (PR) performance metrics. In the next section, the proposed method is described in detail.

## Methods

**Fig. 1:**
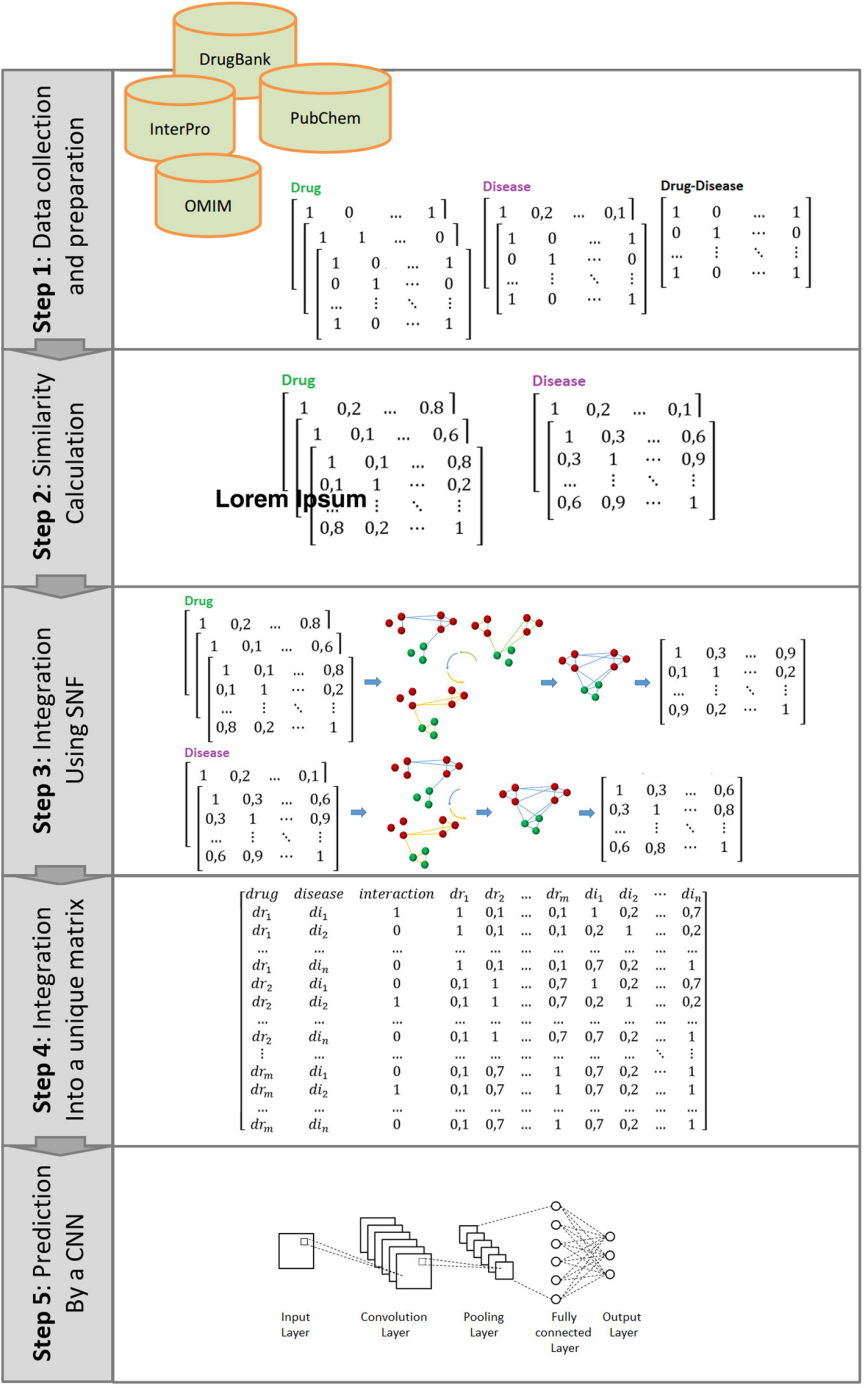
Overview of the proposed approach

The proposed method is elaborated on comprehensively in this section. Figure 1 represents the framework of the method. In the first step, three drug-related matrices and two disease-related matrices as well as a correlation matrix representing the associations between drugs and diseases are prepared as the method input. The Cosine similarity function is used to calculate similarities for drug and disease matrices in step 2. Then, the similarity network fusion (SNF) method is employed to convert the drug and disease similarity matrices into drug and disease similarity matrices in step 3. In the sequel, the drug and disease similarity matrices as well as the drug-disease relationship matrix are merged to construct a unique matrix in step 4. Finally, the constructed matrix is used to train a convolutional neural network which will be utilized to suggest a suitable drug for a target disease in the last step.

### Dataset

To verify IDDI-DNN, the gold standard dataset used for inferring novel drug indications was extracted from the previous research by Gottlieb et al. [28, 30–33]. The dataset contains known drug-disease associations, drug-related properties, and disease-related properties. Drug-related data includes their chemical structure, side effects, and target protein obtained from DrugBank and PubChem. Disease-related data consists of human phenotype and target protein, which are obtained from OMIM and InterPro. Drug-disease associations include 593 drugs approved by the FDA that are within DrugBank [38], and 313 diseases registered in the Online Mendelian Inheritance in Man (OMIM) with 1933 validated DD associations. Both drugs and disease sets have similarities of around 1%. Drug pairwise similarity was calculated using the Tanimoto score [39] as well as disease pairwise calculation using the semantic similarity measure introduced by Slimani [40].

The intended data contains a set of 129,926 samples that are assigned into two classes. The first class consists of 48,724 negative samples indicating that the drug is not suitable for the disease, while the second class includes 81,202 positive samples showing that the drug is appropriate for the disease. To balance the number of positive and negative samples within the dataset, the synthetic minority oversampling technique (SMOTE) was used to generate enough negative samples making the balance rate equal to 0.9.

To further assess the performance of the proposed method, DNdataset was extracted from previous research [41]. DNdataset contains 4,516 diseases annotated by Disease Ontology (DO) terms, 1,490 drugs registered in DrugBank, and 1,008 known drug-disease associations derived from DrugBank.

### The proposed method

#### Definitions

Let us define the set of drugs as $DR={\{dr}_{1},{dr}_{2},\dots {dr}_{m}\}.$ and the set of diseases as $DI={\{di}_{1},{di}_{2},\dots {di}_{n}\}$ where *m* and *n* denote the number of drugs and diseases, respectively. Herein, drug-disease associations called *DR_DI* are represented by a binary matrix $Y\in {R}^{m\times n}$. Each entry ${y}_{ij}$ in this matrix can be 0 or 1, where 1 indicates that drug *i* is suitable for the treatment of disease *j*, and 0 indicates that drug *i* is not suitable for disease *j*. Drug-related similarity is shown by a binary matrix called *DRS* whose elements are displayed as $drs\in {R}^{m\times m}$. Each entry ${drs}_{ij}\in\{0,1\}$ can be 1 indicating that drug *i* is similar to drug *j*, and 0 indicating that drug *i* is not similar to drug *j*. Three *DRS* matrices are created to represent chemical structure, side effects, and target protein for each drug. Disease-related similarity is represented by a binary matrix called *DIS* whose elements are displayed as $dis\in {R}^{n\times n}$. Each entry ${dis}_{ij}\in [0,1]$ can be 1 indicating that disease *i* is similar to disease *j*, and 0 indicating that disease *i* is not similar to disease *j*. Two *DIS* matrices are created to represent human phenotype and target protein for each disease.

$$DR\_DI=\left[\begin{array}{ccccc} & {di}_{1}& {di}_{2}& \dots & {di}_{n}\\ {dr}_{1}& 1& 0& \dots & 1\\ {dr}_{2}& 0& 1& \cdots & 0\\ \dots & \dots & \vdots & \ddots & \vdots \\ {dr}_{m}& 1& 0& \cdots & 1\end{array}\right] DRS=\left[\begin{array}{ccccc} & {dr}_{1}& {dr}_{2}& \dots & {dr}_{m}\\ {dr}_{1}& 1& 0& \dots & 1\\ {dr}_{2}& 0& 1& \cdots & 0\\ \dots & \dots & \vdots & \ddots & \vdots \\ {dr}_{m}& 1& 0& \cdots & 1\end{array}\right] DIS=\left[\begin{array}{ccccc} & {di}_{1}& {di}_{2}& \dots & {di}_{n}\\ {di}_{1}& 1& 0& \dots & 1\\ {di}_{2}& 0& 1& \cdots & 0\\ \dots & \dots & \vdots & \ddots & \vdots \\ {di}_{n}& 1& 0& \cdots & 1\end{array}\right]$$

#### Similarity calculation

The term-frequency vectors are typically very long and, sparse (i.e., they possess many zero values). Several applications use such structures including retrieval of information, clustering text documents, biological taxonomy, and gene feature mapping. The traditional distance measurements do not work well in the case of such sparse numeric data. For instance, two term-frequency vectors may have lots of zero values in common, meaning that the corresponding samples do not share many words. In this study, it is necessary to employ a relevant similarity function that can properly deal with sparse data. Herein, the cosine similarity function was used to calculate the similarity between each pair of drugs in *DRS* matrices and also each pair of diseases in *DIS* matrices. The function calculates the similarity between two vectors using the inner product operation via the formula:

1 $$Cosine\left(x.y\right)=\boldsymbol{ }\frac{{\varvec{x}}.{\varvec{y}}}{\Vert {\varvec{x}}\Vert \Vert {\varvec{y}}\Vert }$$

where ||*x*|| is the Euclidean norm of vector $x=({x}_{1}.{x}_{2}.\dots .{x}_{p})$ and defined as $\sqrt{{x}_{1}^{2}+{x}_{2}^{2}+\dots +{x}_{p}^{2}}$. Conceptually, it is used to calculate the length of a vector. Similarly, ||*y*|| is the Euclidean norm of vector *y*. The measure computes the cosine of the angle between vectors *x* and *y*. A cosine value of 0 means that the angle between two vectors is 90 degree (orthogonal) without any match. The closer the cosine value to 1, the smaller the angle and the greater the match between two vectors [42]. As a result, the values within *DRS* and *DIS* matrices are replaced with calculated similarities in a range of [0, 1].

#### Integration of similarity matrices

The calculated *DRS* matrices (three matrices) are integrated into a unique *DRS* (*UDRS*) matrix using the SNF method. The iterative non-linear process is used by the SNF approach based on message-passing theory for consolidating a given set into one comprehensive matrix [43]. Using the SNF approach, the K-Nearest Neighbors (KNN) algorithm is iteratively applied to update the *UDRS* matrix based on three *DRS* matrices. Similarly, the calculated *DIS* matrices (two matrices) are integrated into a unique *DIS* (*UDIS*) matrix using SNF. Following, two comprehensive similarity matrices, *UDRS* and *UDIS*, for drug and disease similarities integration are represented

$$UDRS=\left[\begin{array}{ccccc} & {dr}_{1}& {dr}_{2}& \dots & {dr}_{m}\\ {dr}_{1}& 1& 0.1& \dots & 0.8\\ {dr}_{2}& 0.1& 1& \cdots & 0.2\\ \dots & \dots & \vdots & \ddots & \vdots \\ {dr}_{m}& 0.8& 0.2& \cdots & 1\end{array}\right] UDIS=\left[\begin{array}{ccccc} & {di}_{1}& {di}_{2}& \dots & {di}_{n}\\ {di}_{1}& 1& 0.3& \dots & 0.6\\ {di}_{2}& 0.3& 1& \cdots & 0.9\\ \dots & \dots & \vdots & \ddots & \vdots \\ {di}_{n}& 0.6& 0.9& \cdots & 1\end{array}\right]$$

#### Merging matrices

Now, three prepared matrices, including *DR_DI*, *UDRS*, and *UDIS*, are merged to construct a new matrix called *F* with m × n rows and m + n + 3 columns as represented following. As a result, the data collected for drugs, diseases, and their associations are integrated into a unique matrix called *F*:

$$F=\left[\begin{array}{ccccccccccc}drug& disease& interaction& {dr}_{1}& {dr}_{2}& \dots & {dr}_{m}& {di}_{1}& {di}_{2}& \cdots & {di}_{n}\\ {dr}_{1}& {di}_{1}& 1& 1& 0.1& \dots & 0.1& 1& 0.2& \dots & 0.7\\ {dr}_{1}& {di}_{2}& 0& 1& 0.1& \dots & 0.1& 0.2& 1& \dots & 0.2\\ \dots & \dots & \dots & \dots & \dots & \dots & \dots & \dots & \dots & \dots & \dots \\ {dr}_{1}& {di}_{n}& 0& 1& 0.1& \dots & 0.1& 0.7& 0.2& \dots & 1\\ {dr}_{2}& {di}_{1}& 0& 0.1& 1& \dots & 0.7& 1& 0.2& \dots & 0.7\\ {dr}_{2}& {di}_{2}& 1& 0.1& 1& \dots & 0.7& 0.2& 1& \dots & 0.2\\ \dots & \dots & \dots & \dots & \dots & \dots & \dots & \dots & \dots & \dots & \dots \\ {dr}_{2}& {di}_{n}& 0& 0.1& 1& \dots & 0.7& 0.7& 0.2& \dots & 1\\ \vdots & \dots & \dots & \dots & \dots & \dots & \dots & \dots & \dots & \ddots & \vdots \\ {dr}_{m}& {di}_{1}& 0& 0.1& 0.7& \dots & 1& 0.7& 0.2& \cdots & 1\\ {dr}_{m}& {di}_{2}& 1& 0.1& 0.7& \dots & 1& 0.7& 0.2& \dots & 1\\ \dots & \dots & \dots & \dots & \dots & \dots & \dots & \dots & \dots & \dots & \dots \\ {dr}_{m}& {di}_{n}& 0& 0.1& 0.7& \dots & 1& 0.7& 0.2& \dots & 1\end{array}\right]$$

#### Neural network architecture

**Fig. 2:**
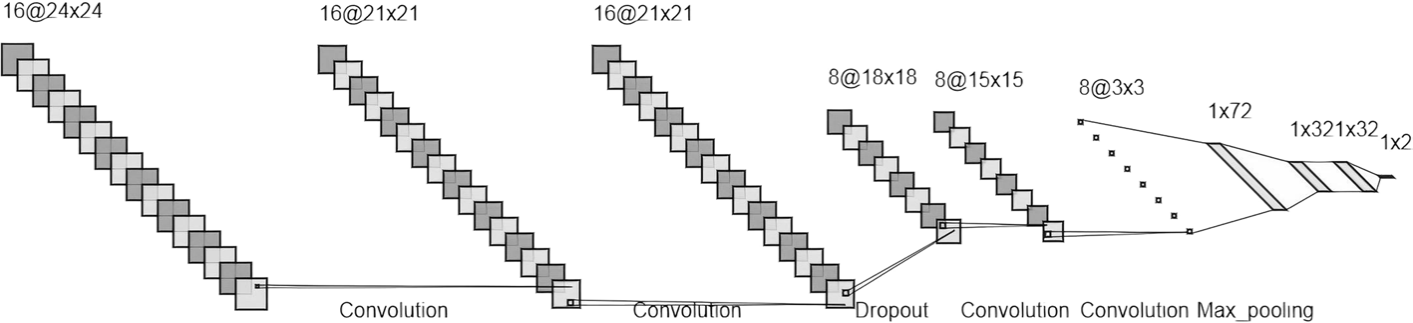
Summary of the CNN architecture

To repurpose a drug and find a new target for disease treatment, a CNN-based model was employed. CNN is a class of artificial neural networks (ANNs) that use deep learning techniques to train its parameters. It is a regularized type of multilayer perceptron whereas its layers are organized purposefully to obtain a high accurate output results. The fundamental structure of CNN contains a convolution layer, a pooling layer, and a fully-connected layer. The convolution layer aims to capture features of the input data to reliably predict the output, while the pooling layer summarizes these features in a low-dimensional vector. The model's hyperparameters and associated values were tuned through several experiments. The best performance was achieved with a CNN having 5 hidden layers each with 300 neurons and a dropout rate of 0.3 for each layer. Regarding that drug-disease association prediction is a binary classification problem, the logistic sigmoid activation function was employed in the output layer, and the binary cross-entropy loss function was used to calculate loss values. The model yields the best results when the Nadam optimization algorithm is used to update weights and bias parameters. The model was fed batch inputs with a batch size of 64. The number of epochs was set to 200 for each run. Figure 2 represents the architecture of the designed CNN after several attempts to tune the structure of the model.

## Results and discussion

### Evaluation criteria

To evaluate the proposed model, fivefold cross-validation has been used. The scheme divides the dataset randomly into five different folds. In each cross-validation, four folds are considered as training sets and the fifth fold is used for testing. The validation is repeated five times randomly, and their average is used to calculate overall variance and bias.

The accuracy of the model was evaluated based on different standard criteria. First, the confusion matrix is calculated based on the predicted outputs of the model. The matrix includes true positive (TP), true negative (TN), false positive (FP), and false negative (FN) predictions. To put things into perspective, TP and TN represent correctly predicted related or unrelated DDs, while FP and FN represent wrongly predicted related or unrelated DDs. Using these four basic metrics from the confusion matrix, Accuracy (Acc), Precision (Prec), Recall (Rec), and F1-score (F1) measures are calculated via the following formulas:

2 $$\mathrm{Acc}= \frac{\mathrm{TP}+\mathrm{TN}}{\mathrm{TP}+\mathrm{TN}+\mathrm{FP}+\mathrm{FN}}$$

3 $$\mathrm{Prec}= \frac{\mathrm{TN}}{\mathrm{TP}+\mathrm{FP}}$$

4 $$\mathrm{Rec}= \frac{\mathrm{TP}}{\mathrm{TP}+\mathrm{FP}}$$

5 $$\mathrm{F}1=2\times \frac{\mathrm{Precision}\times \mathrm{Recall}}{\mathrm{precision}+\mathrm{Recall}}$$

The performance of IDDI-DNN was investigated in comparison to basic machine learning models. Also, the robustness of IDDI-DNN was compared to the latest introduced models for predicting drug-disease associations.

### Training the model

**Fig. 3:**
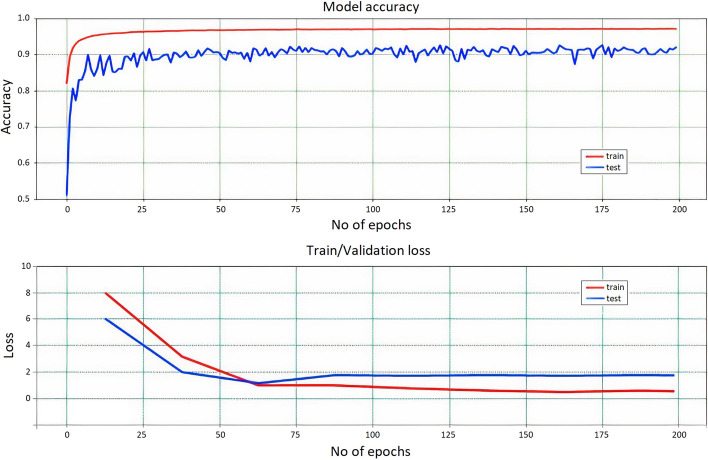
IDDI-DNN training progress in terms of accuracy and loss on training and testing sets

The training progress of IDDI-DNN was screened during the process in terms of accuracy and loss as represented in Fig. 3. To this end, the dataset was divided into 70% of training and 30% of testing subsets. The figure shows that the accuracy of the model on both training and testing data has reached over 95% during the first 20 epochs. This means that the developed deep model is fast enough to reach convergence. In addition, the loss plot of the model indicates that the error rate on both training and testing data is rapidly decreasing, which means that IDDI-DNN reaches its local minimum in a rational time. The trained model is used to predict a drug for a disease. The output of the model for each neuron is in the range between 0 and 1, where 1 indicates the absolute recommendation of an input drug for a disease and 0 indicates the rejection of the drug. The results represent that the density of predictions is mostly zero or one.

### Performance evaluation

The effectiveness of IDDI-DNN was investigated by evaluating its performance in terms of different standard machine learning measures and comparing it to the previously proposed machine learning-based models. The compared methods are decision tree (DT) [44], K-nearest neighbor (KNN) [45], QDA [46], Linear-SVM [47], RBF-SVM [47], and NF-NN [48]. Also, all methods were evaluated using fivefold cross-validation. Table 1 shows the performance of different models in terms of accuracy, precision, recall, and F1-score (top scores are represented in bold). Except for IDDI-DNN, the results for other classifiers were taken from their related works. These results were obtained when the classifiers ran on the gold standard dataset.

**Table 1:** IDDI-DNN performance compared to machine learning-based methods applied on the gold standard dataset

Method	Accuracy	Precision	Recall	F1-score
DT [44]	0.55	0.88	0.12	0.21
KNN [45]	0.65	0.64	0.68	0.66
QDA [46]	0.64	0.64	0.66	0.65
Linear-SVM [47]	0.70	0.70	0.68	0.69
RBF-SVM [47]	0.53	0.70	0.12	0.20
NF-NN [48]	0.79	**0.78**	0.81	0.80
IDDI-DNN	**0.97**	0.69	**0.96**	**0.84**

**Fig. 4:**
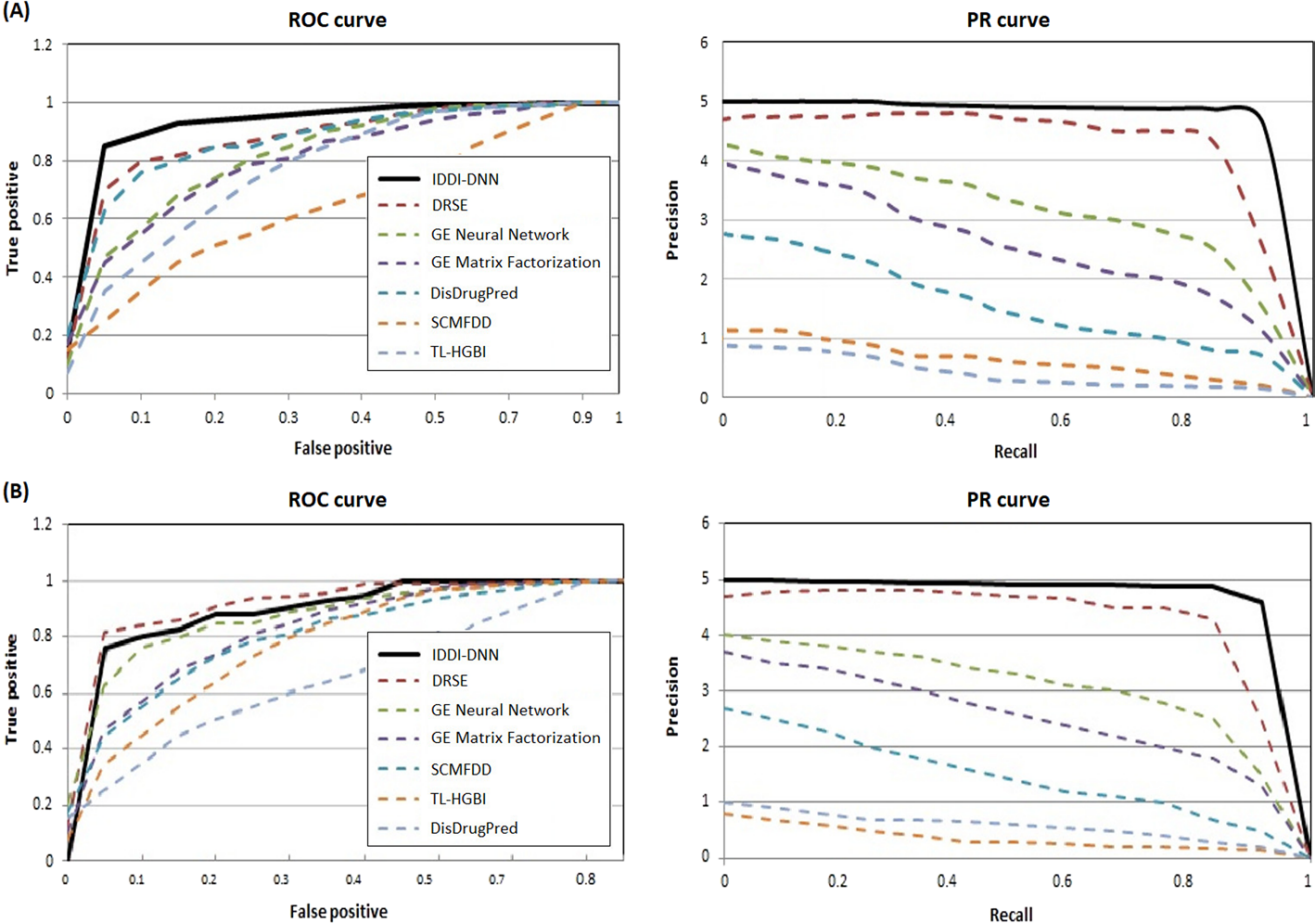
ROC and PR curves obtained by IDDI-DNN and other state-of-the-art methods on (A) the gold standard dataset, and (B) DNdataset

The performance of IDDI-DNN was further assessed in comparison to a number of state-of-the-art methods including SCMFDD [49], TL-HGBI [50], Graph Embedded matrix Factorization [51], Graph embedded neural network [52], DRSE [53], and DisDrugPred [54]. The overall performance of all methods was evaluated by fivefold cross-validation. The experimental results in terms of Receiver Operating Characteristic (ROC) and Precision-Recall (PR) curves are depicted in Fig. 4A.

**Fig. 5:**
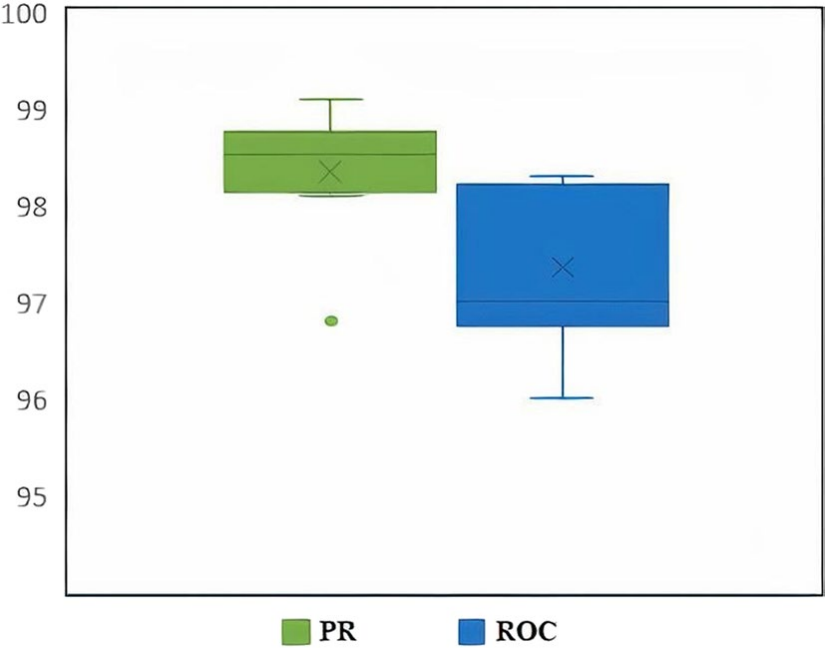
The average PR and ROC obtained by IDDI-DNN after 150 iterations

The robustness of IDDI-DNN was further validated to perform predictions on DNdataset that were used in previous research [41]. After conducting five-fold cross-validation on the DNdataset, the performance of the proposed model was assessed on the dataset. Figure 4B represents the ROC plot of IDDI-DNN in comparison to other state-of-the-art methods on DNdataset. The fivefold cross-validation technique was repeated for 150 rounds and the average results of ROC and PR obtained by IDDI-DNN are shown in Table 2 (top scores are represented in bold). Furthermore, Fig. 5 shows the statistical summary of ROC and PR after 150 rounds in the form of box plots.

**Table 2:** Comparison of IDDI-DNN with other state-of-the-art methods applied to the gold standard dataset

Method	YEAR	ROC	PR
TL-HGBI [50]	2014	72.7	3.0
Graph embedding Matrix Factorization [51]	2015	75.7	69.3
Graph embedding neural network based [52]	2016	77.4	75.2
SCMFDD [49]	2018	63.8	6.0
DisDrugPred [54]	2019	92.0	24.3
DRSE [53]	2021	93.23	94.83
IDDI-DNN	2022	**97.01**	**98.53**

**Fig. 6:**
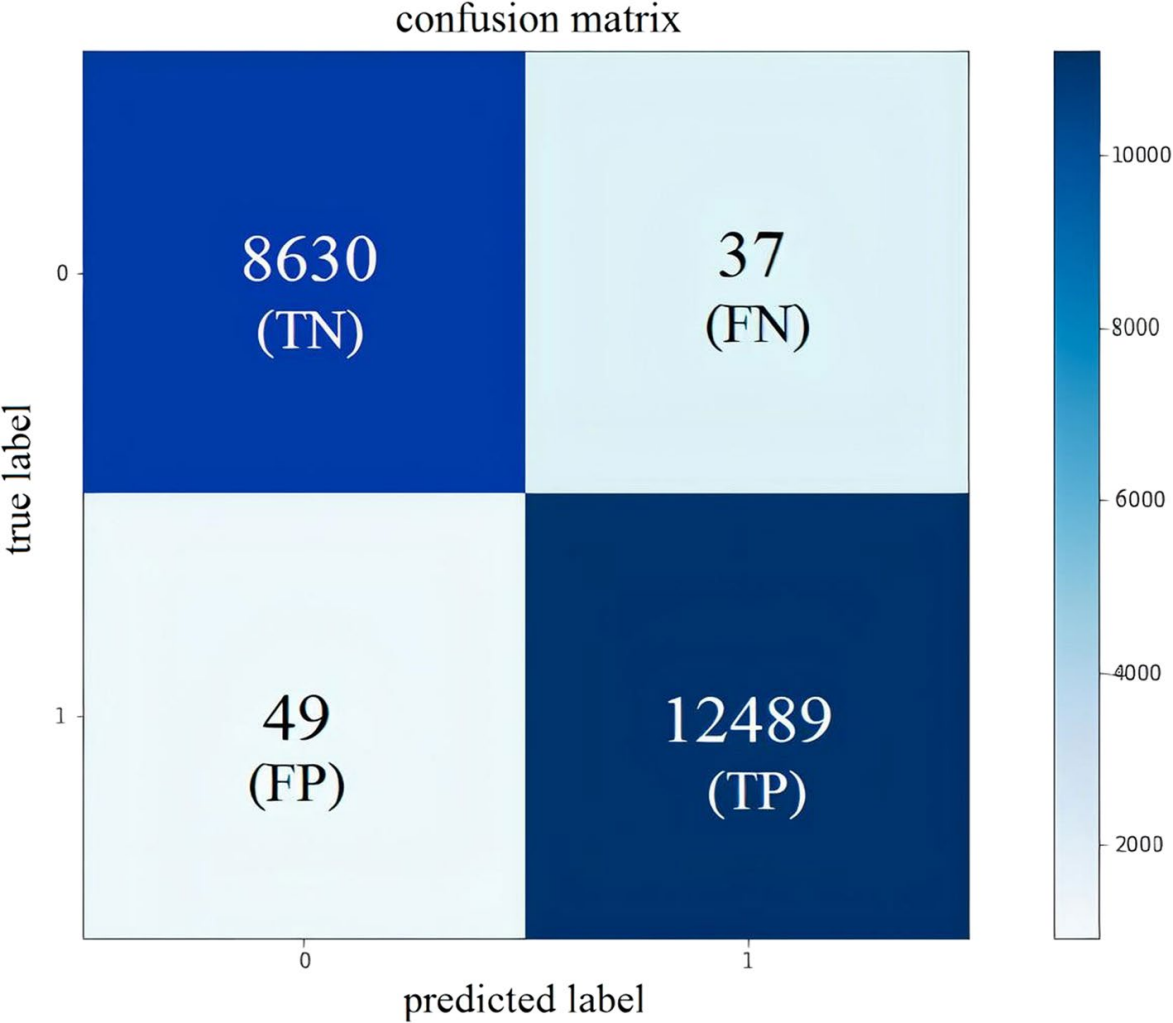
Confusion matrix

The accuracy of IDDI-DNN was further assessed on a number of 21,205 randomly selected data from the gold standard dataset. Figure 6 represents the confusion matrix describing the performance of the model in terms of TP and TN showing the correct predictions of positive and negative associations, and FP and FN showing the incorrect predictions of positive and negative associations.

### Prediction of new drug indications

IDDI-DNN can also be utilized for drugs with no previously known disease association. To this end, we analyzed the performance of all methods for drugs, which has only one known disease association in the golden dataset. In this case, for a given drug, the known associated disease is removed from the dataset, and therefore, the dataset has no associated information for that drug in this experiment. Therefore, the tests for these drugs are used to assess the ability of the method to predict associations for new drugs without known disease association.

**Fig. 7:**
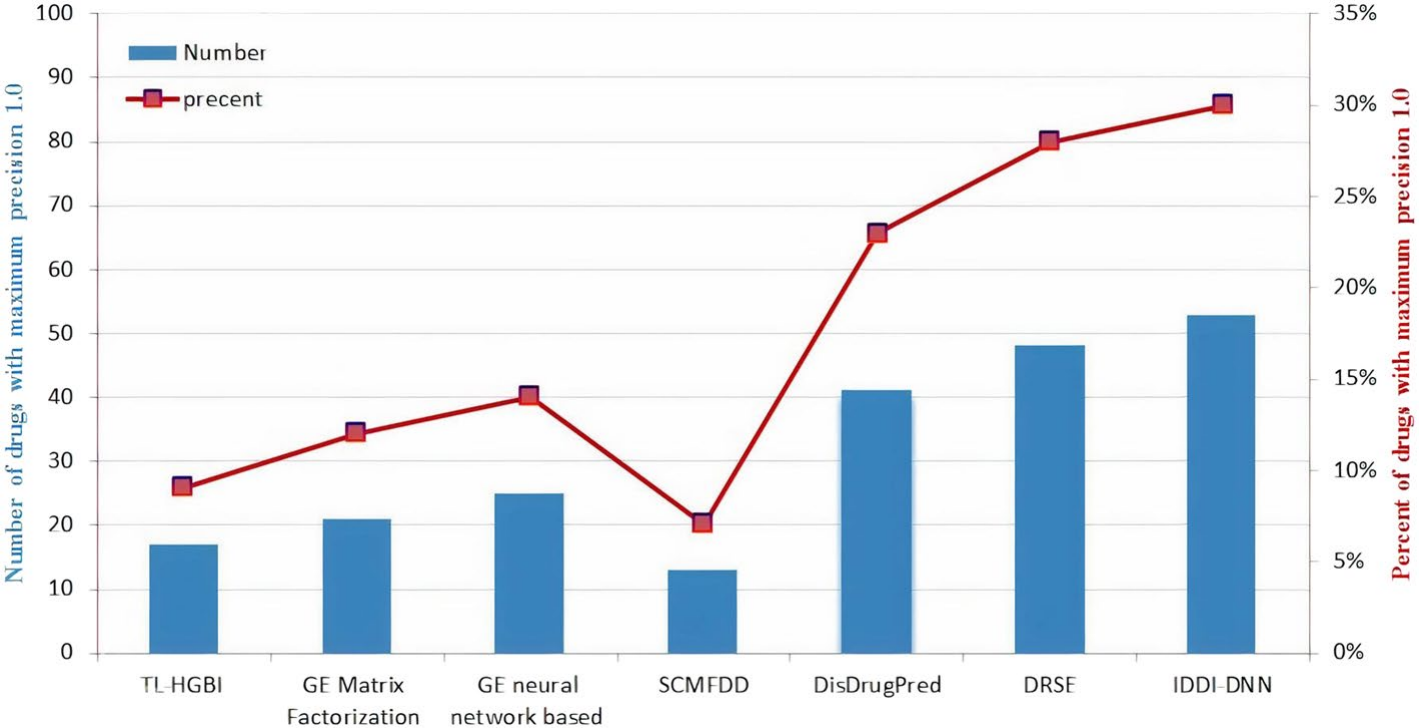
Prediction of new drug indications by different methods with the maximum precision. The results except for IDDI-DNN are from [41]

The gold dataset contains 171 drugs with only one known associated disease. The results shown in Fig. 7 represent the number and percentage of drugs with the maximum precision value of 1.0 obtained by IDDI-DNN and compared to those of other state-of-the-art methods reported in [41]. The maximum precision value of 1.0 means that the disease was successfully ranked as the first candidate disease associated with the particular drug. It can be seen from the figure that IDDI-DNN achieves the best performance among the methods. In addition, 53 out of 171 (30.01%) drugs are predicted with a maximum accuracy of 1.0 by IDDI-DNN. In this experiment, DRSE and DisDrugPred showed 48 (28.31%) and 41 (23.04%) drugs with a maximum accuracy of 1.0, respectively.

### Comprehensive prediction for new drugs

After confirming the predictive ability of IDDI-DNN through cross-validation experiments, we employed the method to predict new associations between all drugs and diseases. In this experiment, all known drug-disease associations in the gold standard dataset were used as the training data, and the remaining drug-disease pairs were considered as potential drug-disease associations.

A case study was conducted to verify whether the predicted disease is correct according to the biological databases including KEGG (https://www.genome.jp/kegg/) and CTD (https://www.ctdbase.org/). These databases contain evidence of drug-disease associations and validation. Furthermore, the DGIdb web server (https://www.dgidb.org) was employed to confirm the results. As a result, new drug-disease associations were predicted by IDDI-DNN and annotated by KEGG and CTD. Among several selected drugs, validated candidate drug-disease associations are represented in Table 3.

**Table 3:** The novel drug-disease associations for the top diseases identified by IDDI-DNN

Disease name	Suggested drug name	Drug accession number	Drug structure
Anal cancer	Capecitabine	DB09037	
Anal cancer	Cisplatin	DB00515	
Anal cancer	Fluorouracil	DB00544	
Stomach ulcer	Berberine	DB04115	
Stomach ulcer	Celecoxib	DB00482	
Stomach ulcer	Rabeprazole	DB01129	
Bacterial Vaginosis	Boric acid	DB11326	
Bacterial Vaginosis	Metronidazole	DB00916	
Chilblains	Secnidazole	DB12834	
Chilblains	Ketoconazole	DB01026	
Insomnia	Solriamfetol	DB14754	
Insomnia	Eszopiclone	DB00402	
Insomnia	Lemborexant	DB11951	

## Discussion

The introduced model, IDDN-DNN, integrates multiple data extracted from different resources for drugs and diseases to accurately repurpose a drug for a disease. In addition to drug-disease association information, the data includes chemical structure, side effects, and target protein for each drug as well as human phenotype and target protein for each disease. This data is integrated into a single matrix *F* and subjected to a CNN-based deep network to train the model. After training, the model is used to repurpose a drug for a target disease.

The proposed model was comprehensively evaluated using two different datasets. First, the gold standard dataset extracted from the previous research by Gottlieb et al. [28, 30–33] was used to examine the performance of IDDI-DNN and compare the results to other machine learning-based methods. The experiments were done using fivefold cross-validation to validate the prediction accuracy. Comparing the results in Table 1, IDDI-DNN obtains a performance of 0.97 and 0.84 in terms of accuracy and F1-score, respectively, higher than other methods.

In another comparative study, the performance of IDDI-DNN was compared to state-of-the-art methods in terms of ROC and PR as represented in Table 2. The results in this table indicate that the proposed method achieves the best score in both measures. In this regard, the PR criterion is a more appropriate measure for evaluating the models because ROC is more sensitive to many zeroes in the association matrix leading to an insignificant increase in this criterion. PR provides more appropriate results by returning known relations, which highlights the capability of the model to predict unrelated DDs. It is obvious from Fig. 4A that IDDI-DNN outperforms other compared methods in terms of ROC and PR. More specifically, IDDI-DNN achieves a ROC of 0.97, while DRSE, DisDrugPred, Graph embedding neural network, Graph embedding Matrix Factorization, TL-HGBI, and SCMFDD obtain inferior results of 0.93, 0.92, 0.77, 0.75, 0.72, and 0.63, respectively. In addition, the PR curve illustrates that IDDI-DNN obtains the best precision against other methods.

In addition, DNdataset was extracted from previous research [41] and used to further validate the robustness of the proposed method. In this experiment, IDDI-DNN achieves a ROC value of 0.82 while DRSE, Graph embedding neural network, Graph embedding Matrix Factorization, SCMFDD, TL-HGBI, and DisDrugPred obtain inferior results of 0.94, 0.79, 0.72, 0.65, 0.36, and 0.28, respectively. The maximum precision achieved by IDDI-DNN is 0.561, which is higher than other methods.

The ability of the IDDI-DNN in the prediction of unknown DD associations was further assessed by removing known associated diseases for all drugs having exclusively one known disease. The results in Fig. 7 demonstrate the preference of IDDI-DNN to other compared state-of-the-art methods. The new DD associations predicted by the method were also investigated using biological databases including KEGG and CTD. The results in Table 3 indicate that the IDDI-DNN predicted associations are valid according to these biological databases.

Given the importance of drug repurposing, different approaches have been proposed for solving various challenges of this issue, such as predicting new drugs, classifying biological data, and analyzing data. In this regard, the learning-based approach is a powerful and widely used solution to make decisions based on existing data. The method introduced by Zhao et al. [36] was recently developed using the learning-based approach. The performance of the drug-disease association model can be improved using recently introduced clustering analysis algorithms such as DBSCAN, Gaussian mixture, mean-shift, and fuzzy approaches. As an example, Hu et al. [55] introduced a fuzzy-based graph clustering algorithm, that increases the prediction performance compared to other state-of-the-art clustering algorithms. In this study, the input data in the form of binary mode (0 indicates the drug is unsuitable for the disease, and 1 indicates the drug is suitable for the disease) is converted to a fuzzy mode ranging between 0 and 1 (the closer the 0, the more unsuitable drug for the disease, and the closer the 1, the more suitable drug for the disease). Furthermore, all prepared matrices (including tree matrices for drugs, two matrices for diseases, and a matrix for drug-disease associations) are merged using the SNF technique. The proposed method for the integration of data prevents information loss and enables the model to accurately repurpose drugs.

## Conclusion

In this research, a novel method for drug repurposing, the so-called IDDI-DNN, is proposed to determine unknown associations between drugs and diseases. In IDDI-DNN, molecular characteristics of drugs as well as disease-related data are extracted from multiple repositories and integrated with the known associations between drugs and diseases. The collected data for drugs, diseases, and their associations are integrated into a unique matrix. The generated matrix is given to a CNN-based model to capture similarities between pairs of drugs and their target diseases and predict potential associations between a drug and a disease. The proposed model was evaluated in terms of accuracy and error rate during the training process. In addition, the robustness and reliability of the method were assessed and compared to the performance of previously introduced methods. The results of assessments demonstrate the preference and applicability of the proposed model in comparison to state-of-the-art drug repurposing methods. The prediction of new drug-disease associations concerning the improvement of known associations is one of the most difficult challenges. IDDI-DNN has proven its superiority to yield fruitful results in this field.

## Data Availability

The primary data used in this research is available via Gottlieb et al. [29]. The rest of the data can be obtained from the corresponding author upon request.
